# Association Between Intra- and Postoperative Opioids in Opioid-Naïve Patients in Thoracic Surgery

**DOI:** 10.1016/j.atssr.2024.04.003

**Published:** 2024-04-23

**Authors:** Kelly L. Wiltse Nicely, Ronald Friend, Chad Robichaux, Jonathan Alex Edwards, Jeannie P. Cimiotti, Kim Dupree Jones

**Affiliations:** 1 Certified Registered Nurse Anesthetist Program, Nell Hodgson Woodruff School of Nursing, Emory University, Atlanta, Georgia; 2 Department of Psychology, Stony Brook University, Stony Brook, New York; 3 Department of Biomedical Informatics, Emory University School of Medicine, Atlanta, Georgia; 4 Georgia Clinical and Translational Science Alliance (CTSA), Atlanta, Georgia; 5 Rollins School of Public Health, Emory University, Atlanta, Georgia; 6 Lincoln International Institute for Rural Health, University of Lincoln, Lincolnshire, UK; 7 Nell Hodgson Woodruff School of Nursing, Emory University, Atlanta, Georgia

## Abstract

**Background:**

As the opioid epidemic continues, a better understanding of the use of opioids in surgery is needed. We examined whether intraoperative opioid administration was associated with greater postoperative opioid use prior to discharge in opioid-naïve patients undergoing thoracic surgery. Further, we sought to determine predictors of higher intra- and postoperative opioid use including demographic and patient factors and hospital.

**Methods:**

Data on patients who underwent elective thoracic surgery between January 1, 2018, and December 31, 2019, were extracted from a data repository at a large health system in the Southeast United States. All patients and data on total intraoperative and postoperative (prior to discharge) opioid administration were included. A total of 126 patient encounters were analyzed.

**Results:**

Increased intraoperative morphine milligram equivalent was associated with increased postoperative administration, where each unit increase in intraoperative morphine milligram equivalent was associated with 0.57 increased units in postoperative use (B = 0.57; 95% CI, 0.29-0.87, *P* < .0003), controlling for patient race, sex, age, weight, Elixhauser comorbidity score, and hospital. Younger age (*P* < .002), comorbidity (*P* < .054), and weight (*P* < .026) were associated with higher intra- and postoperative opioid use, but race (*P* < .320) and sex (*P* < .980) were not associated with opioid administration.

**Conclusions:**

Intraoperative opioid use had a significant impact on postoperative opioid use in patients undergoing elective thoracic surgery, even when controlling for age, weight, comorbidities, race, and sex. Substantial variation in both intra- and postoperative opioid administration was noted.

**In Short**

▪ Intraoperative opioid use had a significant impact on postoperative opioid use in patients undergoing elective thoracic surgery.
▪ There is extensive variability among individuals in the number of opioids administered during thoracic surgery.

Surgery has been identified as a potential contributing factor to the opioid crisis. More than 50 million surgeries are performed each year in the United States, with most patients receiving opioids.^1^ For many people, initial exposure to opioids occurs during surgery. Evidence suggests that opioid-naïve patients who undergo surgery and receive opioids are at risk of developing an opioid use disorder postoperatively.^2^ Nationwide, it has been estimated that 5.9% to 6.5% of patients experience new persistent opioid use after surgery.^3^

A recent study found that 24.4% of patients who were previously opioid-naïve became new, long-term opioid users after undergoing thoracic surgery for cancer.^4^ Similarly, it has been reported that 14% of opioid-naïve patients who underwent thoracic surgery for lung cancer continued to fill opioid prescriptions more than 90 days after surgery.^5^ A comparison of multiple types of cardiothoracic surgery showed that new persistent opioid use after surgery was highest in patients having open lung resection, even after adjusting for multiple patient characteristics. There was also a significant association between the size of the perioperative opioid prescription in oral morphine milligram equivalent (MME) and the rate of new persistent opioid use.^6^

Opioids prescribed or administered outside of the operating room are governed by insurance limits, pharmacy tracking, and prescription drug monitoring programs. However, opioid administration during surgery remains largely outside of these safety nets because intraoperative opioid administration is at the discretion of the anesthesia providers. A recent multicenter study of perioperative outcomes specifically examined the inconsistency in the use of intraoperative opioids, and found substantial variability in opioid use across institutions, even when adjusting for multiple well-described variables.^7^ However, that study was limited to only intraoperative opioid administration, and the authors recommended that pre-, intra- and postoperative opioid measures be assessed and their associations examined.

The risk for opioid-naïve patients to develop new persistent opioid use after surgery may represent a novel target area for impacting the opioid crisis. We hypothesized a positive association between the amount of intra- and postoperative opioid use in opioid-naïve patients undergoing thoracic surgery. Second, we examined potential demographic predictors of intra- and postoperative opioid use such as race, sex, age, weight, hospital, and comorbidity. A final objective examined whether these variables might diminish an intraoperative-postoperative association in opioid administration if such a relationship was observed.

## Patients and Methods

### Study Design and Setting

Expedited approval with waiver of patient consent was granted by the Emory University institutional review board. This retrospective observational study (January 1, 2018, to December 31, 2019) identified patients undergoing elective thoracic surgery based on Current Procedural Terminology codes in 2 academic medical centers in a large Southeast health system. Tracheal, mediastinal, esophageal, diaphragm, cardiac, and aortic procedures were excluded. Current Procedural Terminology codes were included for pneumonectomy, thoracotomy, thoracoscopy, and removal of lung other than pneumonectomy (eg, lobectomy).

A 180-day look-back ensured that patients had not filled an opioid prescription from the health system prior to surgery. Total intraoperative and total postoperative opioid administration prior to discharge were calculated using MME.

### Statistical Analysis

Linear and logistic regressions were conducted to determine whether intraoperative opioid MME was associated with postoperative opioid MME controlling for weight, Elixhauser Index, white vs other race, sex, and hospital setting. Analyses were performed with Statistica 13.3 (StatSoft). Two-tailed *P* < .05 significance and 95% CI were adopted.

## Results

The sample consisted of 129 opioid-naïve patients in the multicenter database that underwent thoracic surgery. Three MME case values were missing. Table 1 provides demographic characteristics for 126 patients with complete data (mean age, 63.3 years; 59.5% white; 43.7% female).

**Table 1:** Demographic and Patient Characteristics

Characteristic	Overall (N = 126)
**Intraoperative opioid, log**	
Median (SD)	0.449 (0.432)
Median (min, max)	0.400 (–0.745, 1.72)
**Sex**	
Female	55 (43.7)
Male	71 (56.3)
**Age**	
Median (SD)	63.3 (15.8)
Median (min, max)	67.4 (23.6, 87.0)
**Race**	
Non-White	51 (40.5)
White	75 (59.5)
**Weight (kg)**	
Median (SD)	85.1 (22.3)
Median (min, max)	80.8 (50.3, 173)
Missing	2 (1.6)
**Hospital**	
Academic medical center 1	77 (61.1)
Academic medical center 2	49 (38.9)
**Elixhauser Score**	
Median (SD)	5.37 (3.54)
Median (min, max)	5.00 (0, 16.0)

Values are presented as n (%) unless otherwise marked.

**Figure 1:**
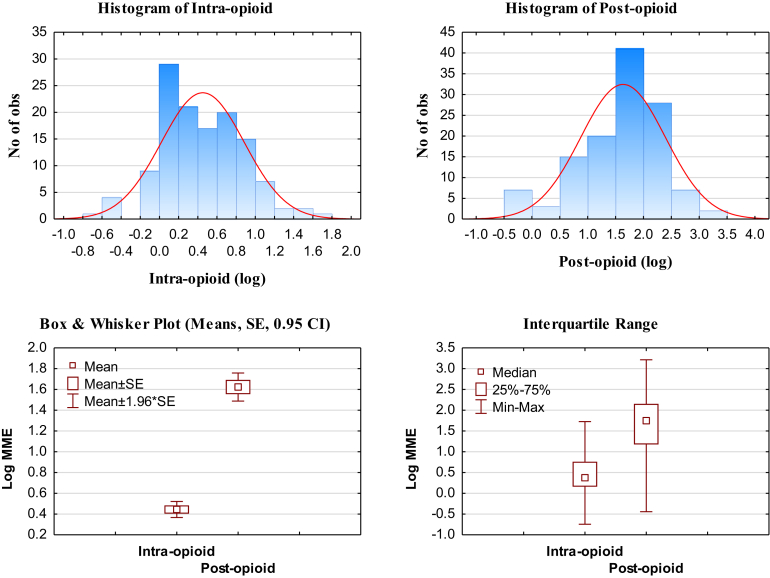
Intra- and postoperative opioid histograms. (CI, confidence interval; MME, morphine milligram equivalent; No of obs, number of observations; SE, standard error.)

Intra- and postoperative opioid MME distributions were positively skewed and therefore log-normal transformed (Figure 1). The mean MME was substantially higher for postoperative than for intraoperative administration (4.69 MME vs 127.57 MME; *P* < .000; Figure 1, lower left); medians (2.42 MME vs 54.72 MME; *P* < .0000; Figure 1, lower right); and interquartile range (1.44-5.76 MME vs 15.00-142.14 MME), showed wide variations.

**Figure 2:**
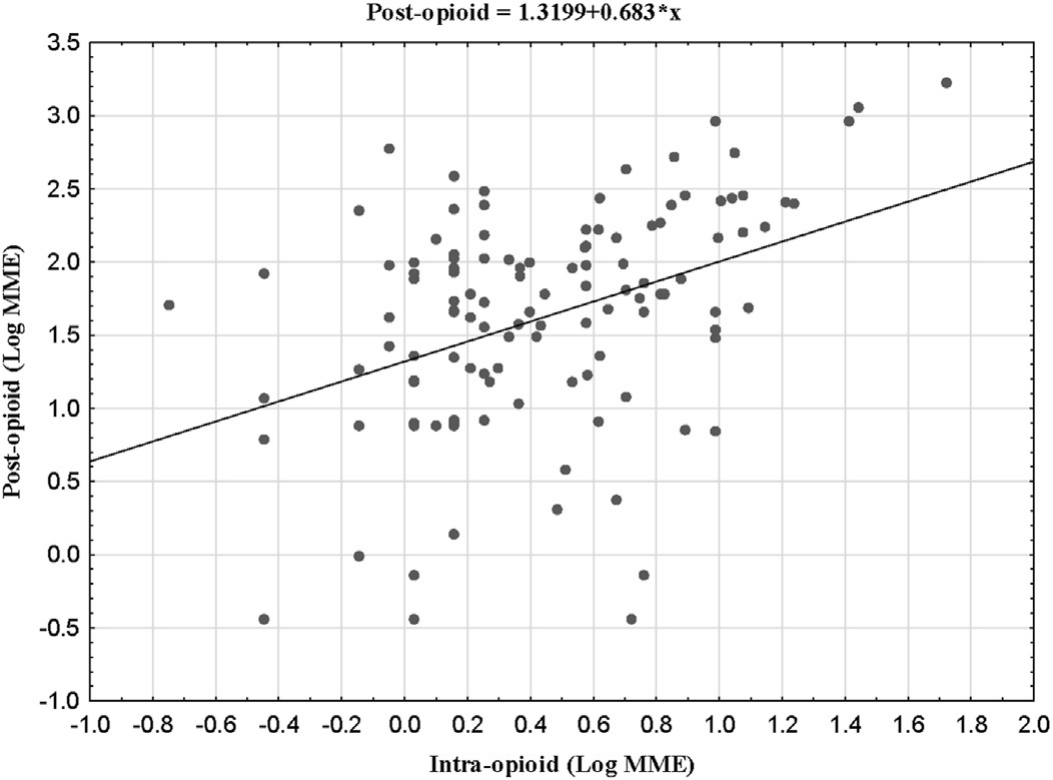
Scatterplot of intra- vs postoperative opioid administration. (MME, morphine milligram equivalent.)

The univariate linear regression showed that 3 of the 7 predictors significantly predicted postoperative opioid administration (Table 2). The strongest predictor was intraoperative opioid administration (Figure 2), showing a 0.68 increase in postoperative opioid administration for every unit of intraoperative administration (b = 0.683; *P* < .0000). Smaller, significant effects were observed for younger age (b = –0.009; *P* < .036) and greater weight (b = 0.007; *P* < .0298). Sex (*P* < .5416), white vs other race (*P* < .5943), and hospital (*P* < .4402) were not significant predictors of postoperative MME.

**Table 2:** Linear and Logistic Regression Showing the Unstandardized (b) and Standardized Coefficient (β) and Logistic Regression (OR) for 7 Predictors of Postoperative Opioid Administration

	Linear Univariate Model (B)^a^	*P* Value	95% CI	Linear Multivariate Model (B)^a^	95% CI	Linear Multivariate Model (β)^b^	*P* Value	Logistic Multivariate (OR)^c^	95% CI	*P* Value
**Intra-Operative Opioid MME**	**0.683**	**<.001**	**0.394 to 0.972**	**0.567**	**0.286 to 0.867**	**0.326**	**<.001**	**1.394**	**1.194 to 1.628**	**<.001**
**Age**	**–0.009**	**.04**	**–0.017 to –0.001**	**–0.011**	**–0.020** to **–0.002**	**–0.233**	**.01**	**0.950**	**0.916 to 0.986**	**.007**
Elixhauser	0.021	.28	–0.016 to 0.058	0.037	–0.001 to 0.076	0.176	.06	1.128	0.966 to 1.317	.13
**Weight**	0.007	**.03**	0.001 to 0.013	0.003	–0.003 to 0.009	0.099	.26	0.955	0.972 to 1.018	.66
Hospital	–0.108	.44	–0.384 to 0.168	–0.063	–0.322 to 0.195	–0.041	.63	0.751	0.259 to 2.174	.60
Sex	0.042	.54	–0.094 to 0.178	0.003	–0.255 to 0.261	0.002	.98	0.712	0.251 to 2.019	.52
White vs Other	0.037	.59	-0.100 to 0.175	-0.130	-0.388 to 0.128	-0.084	.32	1.568	0.539-4.561	.41

Bold font highlights significant values. Linear regression on log-normalized MME (4 log-undefined cases with 0 MME, excluded; n = 122). Logistic regression on MME dichotomized at 75% interquartile point at 142 MME (n = 126).

MME, morphine milligram equivalent.

a Unstandardized coefficient.

b Standardized coefficient.

c Odds ratio.

Multivariate linear regression with the 7 variables found that intraoperative opioid administration remained a strong predictor of postoperative opioid administration (b = 0.567; *P* < .0003) (Table 2), and only slightly reduced (b = 0.683) when controlling for the 6 other variables. Age also remained an independent predictor (b = –0.01; *P* = .01). Weight (*P* = .26), Elixhauser comorbidity score (*P* < .06), hospital (*P* = .063), sex (*P* = .98), and white race (*P* = .32) were not significant.

Logistic regression using raw MME values dichotomized at 142 MME (75% quartile) confirmed the linear regression results. The odds of receiving more than 142 MME postoperatively was 39% higher with increasing intraoperative opioid administration (odds ratio, 1.39; 95% CI, 1.19-1.68; *P* < .0000), controlling for covariates. Again, younger age was associated with greater odds of receiving more than 142 MME (odds ratio, 0.95; 95% CI, 0.92-0.99; *P* = .007). Thus, both the amount of postoperative opioids (linear regression) and the proportion of individuals receiving an amount above the 75% point (logistic regression) were associated with increasing intraoperative opioid administration.

Finally, an internal analysis sought to assess whether those receiving a greater amount of intraoperative MME (above median) showed a stronger pre-post operative association. Pearson r for those above the median was stronger (r = 0.47, *P* < .001; n = 60) than for those below the median (r = 0.24, *P* < .055; n = 62). Overall, the correlation was 0.39 (*P* < .0001).

## Comment

Thoracic surgery is characterized by the least opioid use prior to surgery, but highest rates in new persistent opioid use post-surgery when compared with other surgeries.^8^ A recent article examined the association between intraoperative opioid usage and total amount of opioids administered in the post-anesthesia care unit. Of note, the study found decreased intraoperative opioid administration led to increased opioid requirements in the post-anesthesia care unit.^9^ These findings are in direct opposition to the findings of this study, which considered all opioids administered after surgery until discharge, suggesting that additional investigation is warranted.

A novel feature of our study was identifying intraoperative MME as a critical factor impacting the magnitude of postoperative MME administration in opioid-naïve surgical patients. This association was stronger among those receiving greater amounts of opioid intraoperatively. This intra-post opioid association is important because the magnitude of postoperative MME administered in the immediate 24 hours post-surgery has been found to predict persistent new opioid use.^6^ Reducing intraoperative MME might be a useful adjunct to reducing postoperative opioid use. Such reduction is under the control of the anesthesia provider; it does not require patient education and, when used appropriately, could be easily instituted with benefits postoperatively.^7^^,^^10^

Our findings remained consistent even after controlling for multiple covariates. Only age moderated the intra-post opioid association, but race, sex, weight, and hospital setting did not. Neither was the Elixhauser index, which assesses 29 comorbidities including liver disease and renal failure, a significant factor in changes in opioid use. Despite having a heterogenous mixture of races, our work did not find a significant relationship between patient race and intraoperative or postoperative opioid administration. In a study of 1.1 million patients there were no reported differences in opioid prescribing patterns by race.^7^ However, others have found an association between Black patients and new persistent opioid use after thoracic surgery.^6^ A deeper understanding of the relationship between patient race, intraoperative opioid administration, and persistent long-term postoperative opioid use is necessary.

Our study found extensive variability among individuals in the amount of MME opioids administered. In a large study with more than a million surgical cases across 10 institutions it was reported that there was significant variability in intraoperative opioid use across institutions.^7^ Our study, though limited to thoracic patients in 2 hospitals, shows that this is not only the case intraoperatively (range, 0.18-52.9 MME) but even more so postoperatively (range, 0-1646.5 MME; see also Figure 1). This variability has been noted in all surgical categories, and an institutional-based approach to opioid administration could explain some of the variability.

Data were limited to the intraoperative and immediate postoperative period, with no data available on longer term opioid use or psychosocial factors that are known to impact opioid use. Similarly, we did not assess adjunct medication administration intra- or postoperatively. An important next step will be to assess the association between intraoperative opioid variability and longer-term postoperative outcomes, including the assessment of chronic post-surgical pain.
